# NF-κB signaling in inflammation

**DOI:** 10.1038/sigtrans.2017.23

**Published:** 2017-07-14

**Authors:** Ting Liu, Lingyun Zhang, Donghyun Joo, Shao-Cong Sun

**Affiliations:** 1 Department of Immunology, The University of Texas MD Anderson Cancer Center, Houston, TX, USA; 2 The University of Texas Graduate School of Biomedical Sciences, Houston, TX, USA

## Abstract

The transcription factor NF-κB regulates multiple aspects of innate and adaptive immune functions and serves as a pivotal mediator of inflammatory responses. NF-κB induces the expression of various pro-inflammatory genes, including those encoding cytokines and chemokines, and also participates in inflammasome regulation. In addition, NF-κB plays a critical role in regulating the survival, activation and differentiation of innate immune cells and inflammatory T cells. Consequently, deregulated NF-κB activation contributes to the pathogenic processes of various inflammatory diseases. In this review, we will discuss the activation and function of NF-κB in association with inflammatory diseases and highlight the development of therapeutic strategies based on NF-κB inhibition.

## Introduction

Nuclear factor-κB (NF-κB) represents a family of inducible transcription factors, which regulates a large array of genes involved in different processes of the immune and inflammatory responses.^1^ This family is composed of five structurally related members, including NF-κB1 (also named p50), NF-κB2 (also named p52), RelA (also named p65), RelB and c-Rel, which mediates transcription of target genes by binding to a specific DNA element, κB enhancer, as various hetero- or homo-dimers.^2^ The NF-κB proteins are normally sequestered in the cytoplasm by a family of inhibitory proteins, including IκB family members and related proteins characterized by the presence of ankyrin repeats.^3^ To date, the best studied and most important IκB family member is IκBα. In addition, the precursor proteins of NF-κB1 and NF-κB2, p105 and p100, serve as IκB-like proteins, because their C-terminal potion resembles the structure of IκB and has NF-κB inhibitory functions.^4^

The activation of NF-κB involves two major signaling pathways, the canonical and noncanonical (or alternative) pathways, both being important for regulating immune and inflammatory responses despite their differences in signaling mechanism.^3,5^ The canonical NF-κB pathway responds to diverse stimuli, including ligands of various cytokine receptors, pattern-recognition receptors (PRRs), TNF receptor (TNFR) superfamily members, as well as T-cell receptor (TCR) and B-cell receptor.^6^ The primary mechanism for canonical NF-κB activation is the inducible degradation of IκBα triggered through its site-specific phosphorylation by a multi-subunit IκB kinase (IKK) complex.^1,7^ IKK is composed of two catalytic subunits, IKKα and IKKβ, and a regulatory subunit named NF-κB essential modulator (NEMO) or IKKγ.^8^ IKK can be activated by different stimuli, including cytokines, growth factors, mitogens, microbial components and stress agents.^9^ Upon activation, IKK phosphorylates IκBα at two N-terminal serines and, thereby, triggers ubiquitin-dependent IκBα degradation in the proteasome, resulting in rapid and transient nuclear translocation of canonical NF-κB members predominantly the p50/RelA and p50/c-Rel dimers.^4,7,10^

In contrast to the canonical NF-κB pathway, the noncanonical NF-κB pathway selectively responds to a specific group of stimuli, including ligands of a subset of TNFR superfamily members such as LTβR, BAFFR, CD40 and RANK.^11,12^ In addition, the noncanonical NF-κB activation does not involve IκBα degradation but rather relies on processing of the NF-κB2 precursor protein, p100.^3,11^ A central signaling molecule for this pathway is NF-κB-inducing kinase (NIK), which activates and functionally cooperates with IKKα to mediate p100 phosphorylation, which in turn induces p100 ubiquitination and processing.^13,14^ The processing of p100 involves degradation of its C-terminal IκB-like structure, resulting in generation of mature NF-κB2 p52 and nuclear translocation of the noncanonical NF-κB complex p52/RelB.^3,6,11^ Functionally, canonical NF-κB is involved in almost all aspects of immune responses, whereas the noncanonical NF-κB pathway appears to be evolved as a supplementary signaling axis that cooperates with canonical NF-κB pathway in the regulation of specific functions of the adaptive immune system.^12^

A well-recognized function of NF-κB is regulation of inflammatory responses. In addition to mediating induction of various pro-inflammatory genes in innate immune cells, NF-κB regulates the activation, differentiation and effector function of inflammatory T cells.^15,16^ Recent evidence suggests that NF-κB also has a role in regulating the activation of inflammasomes.^17^ Not surprisingly, deregulated NF-κB activation is a hallmark of chronic inflammatory diseases. Therefore, a better understanding of the mechanism that underlies NF-κB activation and pro-inflammatory function is of great significance for therapeutic strategies in the treatment of inflammatory diseases.

## NF-κB as a mediator of pro-inflammatory gene induction

Inflammation is a protective response of the host to infections and tissue damages, characterized by a series of reactions, including vasodilation and recruitment of immune cells and plasma proteins to the site of infection or tissue injury.^6,15^ Normally, inflammation is beneficial to the host and can be resolved in a timely manner; however, deregulated inflammatory responses can cause excessive or long-lasting tissue damages, contributing to the development of acute or chronic inflammatory diseases. NF-κB is a central mediator of pro-inflammatory gene induction and functions in both innate and adaptive immune cells.

## Function of NF-κB in innate immune cells

Innate immune cells, including macrophages, dendritic cells and neutrophils, are important players of innate immunity and inflammation. These cells express PRRs that detect various microbial components, the so-called pathogen-associated molecular patterns (PAMPs).^18,19^ PRRs also recognize damage-associated molecular patterns (DAMPs), which are molecules released by necrotic cells and damaged tissues. Mammalian cells express five families of PRRs, including toll-like receptors (TLRs), RIG-I-like receptors, NOD-like receptors (NLRs), C-type lectin-like receptors and cytosolic DNA sensors.^18,20,21^ The different families of PRRs have distinct structural properties and respond to different PAMPs and DAMPs, but they share many similarities in the downstream signal transduction pathways.

**Figure 1:**
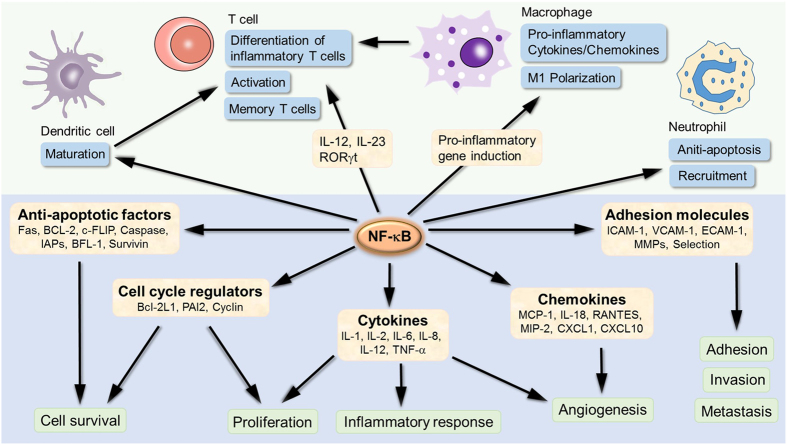
NF-κB target genes involved in inflammation development and progression. NF-κB is an inducible transcription factor. After its activation, it can activate transcription of various genes and thereby regulate inflammation. NF-κB target inflammation not only directly by increasing the production of inflammatory cytokines, chemokines and adhesion molecules, but also regulating the cell proliferation, apoptosis, morphogenesis and differentiation.

A common signaling event of the PRRs is activation of the canonical NF-κB pathway, which is responsible for transcriptional induction of pro-inflammatory cytokines, chemokines and additional inflammatory mediators in different types of innate immune cells (Figure 1).^2,22,23^ These inflammatory mediators can both directly engage in the induction of inflammation and act indirectly through promoting the differentiation of inflammatory T cells. A signaling component that integrates the different PRR pathways for NF-κB activation is transforming growth factor-β-activated kinase 1 (TAK1).^24–26^ TAK1 has two regulatory subunits, TAB1 and TAB2, the latter of which is capable of binding poly-ubiquitin chains, which is required for TAK1 activation. Upon activation, TAK1 activates the downstream kinase IKK, thereby mediating IκBα phosphorylation and NF-κB activation.^27^

The pro-inflammatory function of NF-κB has been extensively studied in macrophages, a large family of innate immune cells that reside in different tissues and function in the front line of an immune response against infections.^28^ In response to diverse PAMPs and DAMPs, macrophages become rapidly activated and secrete a large array of cytokines and chemokines. Under different pathophysiologic conditions, activated macrophages are capable of differentiating into phenotypically different states, including the classically activated (M1) and the alternatively activated (M2) macrophages.^29,30^ M1 macrophages are characterized by the production of pro-inflammatory cytokines, such as IL-1, IL-6, IL-12, TNF-α and chemokines, involved in various inflammatory processes. The M1 macrophages also promote the differentiation of inflammatory T cells, including Th1 and Th17 cells, which in turn mediate inflammation.^30,31^ In contrast, M2 macrophages produce anti-inflammatory cytokines, such as IL-10 and IL-13, and are important for resolution of inflammation and mediating wound healing.^32^ TLR signals have an important role in regulating macrophage polarization.^30^ In particular, the TLR4 ligand lipopolysaccharide (LPS) promotes macrophage differentiation toward M1 phenotype.^24^ LPS stimulates macrophage signaling via two different TLR adapters, MyD88 and TRIF.^24^ Genetic evidence suggests that the MyD88-dependent TLR pathway is crucial for M1 macrophage polarization and inducible expression of pro-inflammatory cytokines.^33^ The MyD88-dependent TLR signaling involves activation of IRAK family of kinases, which in turn stimulate the E3 ubiquitin ligase activity of TRAF6, allowing TRAF6 to undergo self-ubiquitination and to conjugate ubiquitin chains onto other signaling molecules that are involved in the activation of a ubiquitin-dependent kinase, TAK1.^24,25^ Upon activation, TAK1 activates the downstream kinase IKK, which in turn phosphorylate the NF-κB inhibitor IκBα, leading to ubiquitin-dependent IκBα degradation and NF-κB activation.^27^ NF-κB is a key transcription factor of M1 macrophages and is required for induction of a large number of inflammatory genes, including those encoding TNF-α, IL-1β, IL-6, IL-12p40 and cyclooxygenase-2.^30^

A major function of the TRIF-dependent TLR signaling pathway is to mediate the induction of Type I IFNs and IFN-inducible genes.^34^ This pathway involves recruitment of TRAF proteins, particularly TRAF3, to TRIF and subsequent activation of TANK-binding kinase 1 (TBK1) and IKKɛ in a mechanism that is thought to involve TRAF3 ubiquitination and ubiquitin-dependent recruitment of TBK1 and IKKɛ.^34,35^ Activated TBK1 and IKKɛ then phosphorylate the transcription factor IRF3 and, thereby, induce IRF3 dimerization, leading to transcriptional induction of type I IFNs, IFN-α and IFN-β.^30,34^ In addition to IRF3 activation, the TRIF-dependent TLR pathway activates NF-κB though stimulation of the adapter kinase receptor-interacting protein 1 (RIP1).^19,24^ This latter function of TRIF signaling involves activation of an E3 ubiquitin ligase, Peli1, which conjugates lysine 63-linked ubiquitin chains to RIP1, thereby facilitating the recruitment and activation of IKK.^36^ As seen in the MyD88 pathway, TRIF-stimulated NF-κB mediates induction of inflammatory cytokine genes. Therefore, NF-κB is a critical mediator of macrophage inflammatory responses triggered by both the MyD88- and TRIF-dependent pathways. In addition, NF-κB also mediates the pro-inflammatory signaling functions of various other PRRs.

## NF-κB function in T cells

Inflammation also involves adaptive immune components, particularly CD4^+^ T-helper (Th) cells.^37^ The activation of naive T cells is initiated upon engagement of the TCR by a specific antigen presented on antigen-presenting cells, mostly dendritic cells. Canonical NF-κB members, RelA and c-Rel, have a central role in mediating TCR signaling and naive T-cell activation.^38^ Deregulated NF-κB activation can cause aberrant T-cell activation, which is associated with autoimmune and inflammatory responses.^39^ NF-κB also plays a role in regulating T-cell differentiation and effector function. Upon activation, CD4^+^ T cells differentiate into different subsets of effector T cells, including Th1, Th2, Th17 and T follicular (Tfh) cells, which secrete distinct cytokines and mediate different aspects of immune responses.^37^ Th1 and Th17 cells are generally considered as inflammatory T cells, since they mediate inflammatory responses against both infections and self-triggers, and are associated with various autoimmune and inflammatory conditions.^2^ Th1 cells are characterized by the secretion of IFN-γ, a cytokine that both promotes cellular immunity and participate in inflammatory processes. NF-κB promotes Th1 cell differentiation by regulating TCR signaling as well as functioning in innate immune cells to mediate induction of cytokines, such as IL-12, which promote Th1 differentiation.^38^ Th17 cells are characterized by the secretion of IL-17, an inflammatory cytokine that recruits monocytes and neutrophils to the site of inflammation in response to invasion by pathogens or self-antigens. The differentiation of CD4^+^ T cells is regulated by both cytokines secreted by the antigen-presenting cells and other innate immune cells and T-cell intrinsic factors.

Canonical NF-κB regulates CD4^+^ T-cell differentiation via both regulation of cytokine production in innate immune cells and T-cell intrinsic mechanisms. Inhibition of NF-κB in T cells by transgenic expression of a degradation-resistant form of IκBα lacking its N-terminal sequence impairs Th1 responses.^40^ The Th1 cell generation also requires c-Rel, which mainly functions by mediating induction of the Th1-polarizing cytokine in antigen-presenting cells.^41^ NF-κB1 p50, on the other hand, is important for Th2 responses and allergic airway inflammation, which appears to involve induction of the lineage transcription factor Gata3.^42^ Several NF-κB members have been shown to promote Th17 responses. *Nfkb1* knockin mice that express p50 but not its precursor, the IκB-like molecule p105, display aberrant NF-κB activation and spontaneously develop colitis characterized by hyperproduction of Th17 cells.^43^ Although p105 deficiency has no T-cell intrinsic effect on Th17 cell differentiation, the aberrant activation of NF-κB renders innate immune cells hyperresponsive to TLR stimulation for production of IL-6, a major cytokine-promoting Th17 differentiation.^43^ A T-cell intrinsic role of NF-κB in regulating Th17 responses was initially indicated by a finding that mice with T-cell-specific IKKβ deletion have impaired T-cell activation and are refractory to the induction of a Th17-dependent autoimmune disease, experimental autoimmune encephalomyelitis (EAE).^44^ Subsequent work has definitively demonstrated a crucial role for c-Rel and RelA in mediating induction of the Th17 lineage transcription factor RORγt and the generation of Th17 cells.^40,41^ In CD4^+^ T cells, c-Rel also mediates TCR-stimulated expression of IL-21, a γc family cytokine important for the differentiation Th17 and Tfh cells.^45^ Consistently, the c-Rel-deficient mice have a defect in both Th17 and Tfh responses.^45^

Regulatory T (Treg) cells, generated along with thymocyte development or through CD4^+^ T-cell differentiation, are instrumental for controlling immune responses to prevent autoimmunity and chronic inflammation.^46^ Although NF-κB is known as a factor that promotes T-cell activation and effector T-cell differentiation, it is increasingly clear that the function of NF-κB in T-cell responses is paradoxical, since it is also involved in the generation of Treg cells. Mice deficient in various signaling components of the canonical NF-κB pathway, such as TAK1, IKK and the T-cell-specific TAK1/IKK-activating factors CARMA1 and Bcl10, have reduced production of Treg cells, whereas expression of a constitutive active IKKβ or deletion of the IKK-negative regulator CYLD promotes Treg development.^47^ The NF-κB member c-Rel is particularly important for mediating Treg development, and c-Rel acts by participating in the induction of Treg master transcription factor Foxp3.^48,49^ The canonical NF-κB signaling pathway is also required for maintaining the immunosuppressive function of Treg cells, since deletion of IKKβ or its upstream activator Ubc13 in Treg cells impairs the *in vivo* function of Treg cells and sensitizes Treg cells for acquiring Th1 and Th17 inflammatory effector functions under lymphopenic conditions.^50^

Although noncanonical NF-κB pathway is dispensable for naive T-cell activation, this pathway is required for both the differentiation and effector/memory functions of T cells, as demonstrated using different *in vivo* models of immune and autoimmune responses.^51–54^ Mutant mice harboring NIK gene deletion or expressing a non-processible p100 displays impaired generation of Th1 and Th17 subsets of CD4^+^ effector T cells. NIK and noncanonical NF-κB are also required for the recall responses of antigen-specific effector and memory T cells.^51–54^ Moreover, noncanonical NF-κB is required for the pathological effector function of Th17 cells in mediating neuroinflammation, which involves induction of the inflammatory cytokine GM-CSF.^53^ Of note, in contrast to its *in vivo* role in regulating Th1 and Th17 effector T-cell generation, noncanonical NF-κB pathway is dispensable for CD4^+^ T-cell differentiation in an *in vitro* system involving naive T-cell activation with anti-CD3/anti-CD28 in the presence of polarizing cytokines.^52,53^ This difference is likely due to the requirement of *in vivo* conditions for optimal activation of noncanonical NF-κB. As indicated above, noncanonical NF-κB activation is primarily mediated by a subset of TNFR superfamily members. T-cell activation is associated with inducible expression of several TNFRs, including CD27, CD30, OX40 and 4-1BB, which are engaged by their ligands on antigen-presenting cells.^55^ Although some of the TNFR ligands are also expressed on activated T cells, the T cell–T cell interaction, especially under *in vitro* conditions, only trigger weak activation of noncanonical NF-κB, which can be greatly enhanced by cross-linking the TNFR OX40.^51,53^ Collectively, these findings suggest that both canonical and noncanonical NF-κB pathways are involved in the generation and effector functions of inflammatory T cells, although they differ in mechanisms of activation and function.

## NF-κB in inflammasome regulation

**Figure 2:**
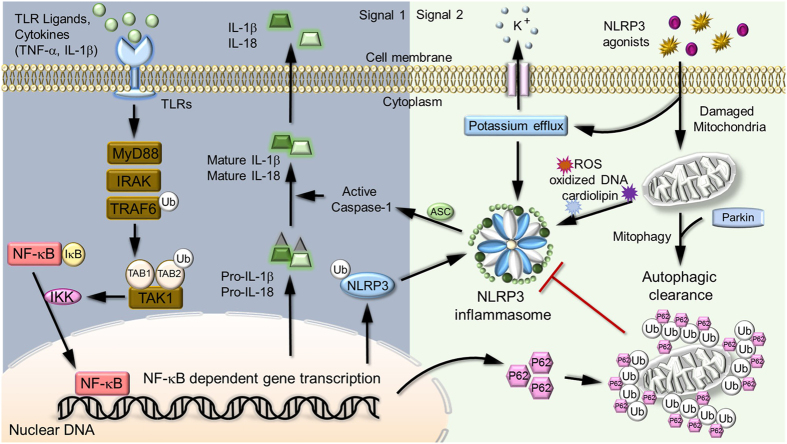
NF-κB in the regulation of NLRP3 inflammasome. The activation of NLRP3 inflammasome requires two signals, priming and activation. A prototypical example of priming is bacterial LPS binding to TLR4, leading to the activation of NF-κB signaling. In the nucleus, the active NF-κB promotes the transcription of NF-κB-dependent genes, such as NLRP3, Pro-IL-1β and Pro-IL-18, which are necessary for inflammasome activation. The second signal of inflammasome activation is provided by NLRP3 agonists that activates NLRP3 to trigger inflammasome assembly and mature IL-1β secretion. To date, mitochondrial damage is the most widely studied activating stimuli for NLRP3 pathway in terms of its connection to diverse inflammatory, metabolic and malignant diseases. NF-κB induces delayed accumulation of the autophagy receptor p62, which can specifically bind to mitochondrial poly-ubiquitin chains though E3 ubiquitin ligase Parkin, and thereby, negatively regulate inflammasome activation via mitophagic elimination.

Inflammasomes are a group of intracellular multi-protein complexes assembled in response to PAMPs and DAMPs, and characterized by the activation of inflammatory caspases.^21^ Canonical inflammasomes are composed of a ligand-sensing receptor, which includes members of the NLR family as well as AIM2 (absent in melanoma 2), the adapter protein ASC (apoptosis-associated speck-like protein containing CARD) and pro-caspase 1.^56^ Among the well-characterized inflammasome receptors are NLRP1, NLRP3, NLRC4 and AIM2. Upon stimulation, the inflammasome receptors oligomerize and recruit pro-caspase 1 via ASC, thereby stimulating pro-caspase 1 processing and conversion to active caspase 1. Activated caspase 1 then cleaves pro-IL-1b and pro-IL-18 into their mature forms, leading to the secretion of these pro-inflammatory cytokines.^57^ Inflammasomes form an integral part of the innate immunity against pathogenic infections and also play an important role in regulating the composition of intestinal microbiota.^58^ However, deregulated inflammasome activation contributes to various autoimmune and inflammatory diseases.^59^ It is now clear that NF-κB signaling pathway is involved in the regulation of inflammasome, contributing to the initiation and development of inflammatory diseases (Figure 2).^59^

NLRP3 inflammasome is currently the most extensively studied inflammasome, which is composed of NLRP3, ASC and pro-caspase 1, as well as an essential regulatory protein, NIMA-related kinase 7 (NEK7). Activation of the NLRP3 inflammasome usually requires both a priming signal (signal 1) and an activation signal (signal 2). A major role of the priming signal is to induce the transcriptional expression of NLRP3 and pro-IL, since most cell types have insufficient levels of NLRP3 for inflammasome activation and do not constitutively express pro-IL-1β.^60,61^ In addition, emerging evidence suggests that signal 1 may also prime NLRP3 via post-translational mechanisms, such as NLRP3 deubiquitination.^62,63^ Typical inducers of signal 1 include microbial components, such as TLR ligands, and cytokines like TNF-α and IL-1β, which are known to activate NF-κB, a transcriptional activator of both *NLRP3* and *pro-IL-1β* genes. The second signal of inflammasome activation is triggered by various PAMPs and DAMPs, such as pore-forming toxins, viral RNAs, ATP and crystalline substances.^60,64^ These diverse stimuli are thought to activate NLRP3 via inducing different cellular events, including K^+^ efflux, Ca2^+^ signaling, mitochondrial and lysosomal damages that release substances such as reactive oxygen species, oxidized mitochondrial DNA and lysosomal proteases.^61^

NF-κB is a central mediator of the priming signal of NLRP3 inflammasome activation and acts by inducing the transcriptional expression of NLRP3 and pro-IL-1β in response to various PRR ligands and cytokines.^3,17^ Like the *pro-IL-1β* gene, the *NLRP3* gene is a direct target of NF-κB and contains NF-κB-binding sites in its promoter region.^65^ Thus, incubation of LPS-stimulated macrophages with an IKK inhibitor, Bay11-7082, blocks NLRP3 induction and caspase 1 activation by ATP.^57,66^ However, the role of NF-κB signaling pathway in inflammasome regulation appears to be complex, since IKKβ-deficient macrophages display hyper-activation of caspase 1 and enhanced secretion of IL-1β upon LPS stimulation, and myeloid cell-conditional IKKβ knockout mice are more sensitive to endotoxin shock.^67^ The negative role of IKKβ in inflammasome activation appears to involve induction of autophagy, an intracellular degradation system that maintains cellular homeostasis through degradation of abnormal proteins and damaged organelles like mitochondria.^68^ Earlier studies suggest that IKK is important for induction of autophagy, which in turn negatively regulates inflammasome activation by maintaining healthy mitochondria to prevent release of reactive oxygen species mitochondrial DNA and possibly also by degrading major components of the inflammasome complex.^69–72^ IKK/NF-κB facilitates autophagy induction by inducing the expression of an autophagy receptor, p62 (also called SQSTM1), mediating recruitment of damaged mitochondria for autophagic clearance via a ubiquitin-dependent mechanism.^68^ Myeloid cell-specific p62 ablation results in aberrant accumulation of damaged mitochondria and excessive production of IL-1β, associated with hyper-sensitivity to endotoxin-induced shock. Collectively, these findings suggest although NF-κB mediates the priming signal of NLRP3 inflammasome activation, induction of p62 expression and mitophagy by NF-κB may serve as an autoregulatory mechanism to restrain its pro-inflammatory function.

## NF-κB in inflammatory diseases

NF-κB has been implicated in the pathogenesis of a number of inflammatory diseases, such as rheumatoid arthritis (RA), inflammatory bowel disease (IBD), multiple sclerosis, atherosclerosis, systemic lupus erythematosus, type I diabetes, chronic obstructive pulmonary disease and asthma.^73^ In response to different cellular stimuli, NF-κB plays a complex role in different cell types and in different diseases states.

### Rheumatoid arthritis

RA is an autoimmune and inflammatory disease characterized by immune cell infiltration into the synovium, associated with chronic inflammation and destruction of cartilage and bone.^74^ A major inflammatory mediator of RA is NF-κB, which has been demonstrated in studies using both animal models and human patients. For example, several early studies have detected NF-κB activation in synovial tissue of RA patients.^75–78^ In mouse collagen-induced arthritis, NF-κB activation in synovial tissue precedes the development of clinical symptoms and increases along with disease progression.^79^ NF-κB activation has also been associated with rat arthritis induced by different agents, such as pristine and streptococcal cell wall.^80,81^ Similarly, NF-κB activation in rats by intra-articular transfer of an adenoviral vector encoding IKKβ induces synovial inflammation and clinical signs of arthritis, whereas intra-articular transfer of a dominant-negative IKKβ mutant suppresses adjuvant-induced arthritis.^82^ In line with these findings, mice with myeloid cell-specific deficiency of A20, a deubiquitinase negatively regulating NF-κB signaling, spontaneously develop polyarthritis with typical features of RA.^83^ Finally, NF-κB inhibition by decoy oligonucleotides or the IKK inhibitor BMS-345541 ameliorates adjuvant-induced arthritis.^16,84^

The pathogenesis of RA involves a variety of cell types, including innate immune cells such as monocytes/macrophages, T cells, B cells and synovial fibroblasts.^85^ NF-κB mediates the induction of pro-inflammatory cytokines, such as TNF-α, IL-1 and IL-6, in monocytes/macrophages.^86^ Many of these cytokines are capable of activating NF-κB in innate immune cells and fibroblasts, thereby inducing the expression of additional inflammatory cytokines and chemokines, leading to further recruitment of inflammatory immune cells and dissemination of inflammation.^84^ The canonical and noncanonical NF-κB pathways also mediate RANK ligand-induced differentiation of monocytes/macrophages into the bone-resorbing osteoclasts, whose deregulation contributes to inflammatory bone loss associated with RA.^87–89^ Among the different subsets of T cells, Th17 cells are particularly important for the pathogenesis of RA.^90^ As described above, NF-κB promotes Th17 differentiation both indirectly through induction of inflammatory cytokines, IL-1, IL-6 and IL-23, in innate immune cells and directly regulates Th17 lineage transcription factors in T cells.^2,91,92^ Deregulated activation of NF-κB also contributes to aberrant survival of self-reactive B cells and production of auto-antibodies that contribute to the pathogenesis of RA.^93^ In particular, RA patients often display elevated serum levels of B-cell activating factor belonging to TNF family associated with deregulated activation of the noncanonical NF-κB. Therefore, NF-κB mediates the pathogenesis of RA by functioning in different cell types.

### Inflammatory bowel disease

Inflammatory bowel diseases, including Crohn’s disease and ulcerative colitis, are chronic inflammatory disorders of the gastrointestinal tract thought to result from inappropriate inflammatory responses to intestinal microbes.^94^ The pathogenesis of IBD involves multiple cell types of the mucosal immune system, including intestinal epithelial cells, innate immune cells such as macrophages and neutrophils, T cells and innate lymphoid cells.^95^ Strong evidence suggests the involvement of NF-κB in the pathogenesis of IBD. Constitutive NF-κB activation has been found in inflamed colonic tissue of IBD patients.^96,97^ Furthermore, genetic mutations in NF-κB-stimulating immune receptors, such as NOD2, and NF-κB target genes, such as IL-12 and IL-23, are associated with human IBD.^94^ Polymorphisms and mutations in the *NFKB1* gene, which encodes the IκB-like molecule p105 and its processing product p50, have also been associated with IBD.^98–100^ These genetic alterations appear to inhibit *NFKB1* gene expression or alter the stability and function of the protein products. Consistently, mice carrying a knockin mutation in the *NFKB1* gene to block generation of p105 spontaneously develop intestinal inflammation with IBD-like features.^43^ A number of other animal model studies have also demonstrated that genetic deficiency in negative regulators of the canonical NF-κB pathway, such as the deubiquitinases CYLD and A20, promotes colonic inflammation.^101–103^ In line with these findings, decoy oligonucleotides that target the DNA-binding activity of NF-κB proteins ameliorate colitis induced by trinitrobenzene sulfonic acid and Dextran sulfate sodium.^104,105^ Deletion of IKKβ in myeloid cells also inhibits experimental colitis and colitis-associated cancer.^106^ These findings are consistent with the role of NF-κB in mediating induction of pro-inflammatory cytokines in innate immune cells and the differentiation Th1 and Th17 subsets of inflammatory T cells.

In contrast to its pro-inflammatory role in myeloid cells, NF-κB has a protective role in intestinal epithelial cells, where it is required for maintaining epithelial integrity and intestinal immune homeostasis.^107,108^ Conditional deletion of NEMO, IKKβ or both IKKα and IKKβ in intestinal epithelial cells causes spontaneous development of chronic intestinal inflammation in mice.^107,108^ Thus, aberrant activation of NF-κB or its genetic deficiency may both contribute to the pathogenesis of IBD, with its functions differing between innate immune cells and epithelial cells.

### Multiple sclerosis

Multiple sclerosis is an inflammatory disease of the central nervous system (CNS) generally considered to be an autoimmune disease involving the pathogenic action of CNS-specific CD4^+^ T cells, particularly Th1 and Th17 cells.^109^ The involvement of NF-κB signaling pathway in multiple sclerosis has been suggested by genome-wide association studies. These studies have identified a number of NF-κB-related factors as susceptibility candidates, such as RelA, IκBα, IκBz, NIK, Bcl10 and MALT1.^110–112^ Consistently, both the canonical and noncanonical NF-κB pathways play an important role in the pathogenesis of EAE, a widely used animal model of multiple sclerosis involving immunization of mice with peptides derived from CNS proteins, such as myelin oligodendrocyte glycoprotein.^2,113^. T-cell-specific deletion of IKKβ or oral administration of an IKKβ inhibitor, PS1145, renders mice refractory to EAE induction.^44^ Genetic deficiency in IKK upstream signaling factors of the TCR pathway, including CARMA1 and MALT1, also ameliorate EAE induction.^114–116^ The canonical NF-κB members RelA and c-Rel mediate expression of the Th17 lineage transcription factor RORγt and, thereby, promote Th17 differentiation.^117,118^ An important role of noncanonical NF-κB pathway in EAE regulation has been demonstrated using mutant mice lacking the kinase NIK or expressing a processing-defective p100 mutant.^52,53,119,120^ Noncanonical NF-κB regulates both recall responses and encephalitogenic function of Th17 cells.^52,53^ Regarding the latter, the noncanonical NF-κB member p52, in synergy with c-Rel, mediates expression of the inflammatory cytokine GM-CSF in Th17 cells.^53^

In addition to its function in regulating the differentiation and effector function of T cells, NF-κB regulates EAE through action of innate immune cells. Constitutive activation of NF-κB in myeloid cells, as a result of IκBα deletion using the LysM-Cre system, causes more severe CNS inflammation and clinical scores in myelin oligodendrocyte glycoprotein-induced EAE,^121^ whereas myeloid cell-specific deletion of IKKβ inhibits EAE induction associated with impaired generation of inflammatory Th1 and Th17 cells.^122^ NF-κB also functions in the CNS to regulate neuroinflammation, since conditional deletion of NEMO or IKKβ using Nes-Cre, which is specific for neuronal cells including neurons, astrocytes and oligodendrocytes, partially inhibits EAE induction.^123^ Furthermore, NF-κB inhibition in astrocytes via transgenic expression of a degradation-resistant form of IκBα (IκBα-dn) inhibits inflammatory cytokine expression and reduces the disease severity in EAE.^124,125^

### Atherosclerosis

Atherosclerosis is a progressive and inflammatory disorder of the arterial wall, characterized by the accumulation of low-density lipoprotein (LDL) particles and immune cells in the subendothelial space.^126^ The pathogenesis of atherosclerosis involves different cell types, including endothelial cells, monocytes and T cells.^126^ It is generally thought that the disease initiation involves activation of endothelial cells to express chemotactic factors and cell adhesion molecules that mediate recruitment of blood monocytes into the arterial intima, where they differentiate into macrophages and, following uptake of LDL particles, eventually become lipid-laden foam cells involved in atherosclerotic plaque formation. NF-κB regulates the expression of a large array of genes involved in different aspects of atherosclerotic pathogenesis.^127^ In vascular endothelial cells, NF-κB mediates induction of pro-inflammatory cytokines, chemotactic factors and adhesion molecules, thereby promoting monocyte recruitment and disease progression.^127–131^ Conditional deletion of NEMO or transgenic expression of a degradation-resistant IκBα in endothelial cells inhibits chemokine expression and monocyte recruitment, coupled with reduced disease severity of atherosclerosis, in ApoE-deficient mice fed with a cholesterol-rich diet.^129^ NF-κB also functions in myeloid cells to promote inflammatory gene expression and conversion of macrophages into foam cells.^127^ Transgenic expression of a non-degradable IκBα in macrophages reduces lipid loading and foam-cell formation, whereas myeloid cell-specific IκBα deletion sensitizes atherosclerosis development in LDL receptor-deficient mice.^132,133^ In line with these findings, myeloid cell-specific deletion of IKKβ reduces atherosclerotic lesion areas in LDL receptor-deficient mice fed with high-fat diet, which is associated with attenuated activities of macrophages in inflammatory gene expression, adhesion, migration and lipid uptake.^134^ Surprisingly, however, an earlier study suggests that deletion of IKKβ in myeloid cells increases atherosclerotic lesion sizes in the LDL receptor-deficient mice.^134,135^ The reason for such a discrepancy is unclear, although it could be due to the differential experimental approaches used in these two different studies.

## Concluding remarks

**Figure 3:**
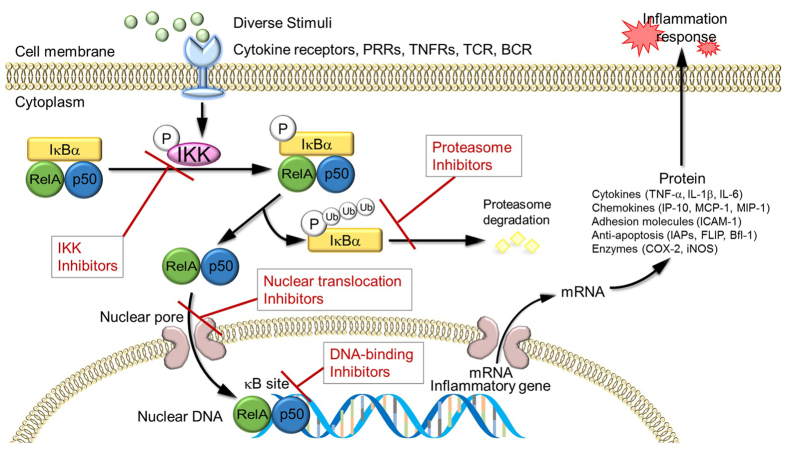
NF-κB-targeted therapeutics in inflammatory diseases. NF-κB signaling plays a pathogenic role in various inflammatory diseases; therefore, there are many therapeutic strategies for inflammatory diseases aimed at blocking NF-κB activity. First, inhibition of IKK kinase activity. Drugs such as aspirin and salicylate have the ability to specifically inhibit IKK, thereby preventing phosphorylation of IκBα. Second, inhibition of protease activity. Drugs such as PS-341 and lactacystin specifically inhibit 26S proteasome complex, thereby preventing IκBα degradation. Third, inhibition of nuclear translocation. Drugs such as tacrolimus and IκBα super-repressor specifically prevent NF-κB subunits RelA, p50, c-Rel and other members from entering the nucleus. Finally, inhibition of DNA binding. Drugs such as glucocorticoids and PPAR agonists have the ability to prevent NF-κB subunits from binging with target genes, and therefore inhibit the transcription.

It is now well accepted that NF-κB serves as a central inflammatory mediator that responds to a large variety of immune receptors. Since deregulated NF-κB activation is involved in various inflammatory diseases, targeting the NF-κB signaling pathway represents an attractive approach for anti-inflammatory therapies. Several categories of inhibitors have been developed to block different steps of NF-κB signaling (Figure 3). (1) An increasing number of selective IKK inhibitors have been designed to block the catalytic activity of IKK and prevent IκBα phosphorylation.^136^ Some well-known anti-inflammatory drugs, such as aspirin and salicylate, also have the ability to inhibit IKK.^137^ (2) Proteasome inhibitors, such as Velcade (also called Bortezomib and PS-341) and lactacystin, which block IκBα degradation in the proteasome. (3) Inhibitors that block nuclear translocation of different NF-κB subunits, such as tacrolimus (FK-506) and IκBα super-repressor. (4) Drugs that inhibit the DNA-binding activity of NF-κB, such as glucocorticoids and PPAR agonists. However, while significant progress has been made in designing approaches to inhibit NF-κB, complexities exist for the development of clinically available NF-κB-based drugs. Although NF-κB inhibition could be beneficial in treating inflammatory diseases, there are obvious questions regarding the balance between efficacy and safety, since NF-κB function is also required for maintaining normal immune responses and cell survival. Accumulating studies suggest that global inhibition of NF-κB signaling may cause severe side effect. Therefore, better understanding of the mechanism underlying the pathological activation of NF-κB in individual diseases is crucial for designing more specific and effective therapeutic agents for the treatment of inflammatory diseases.
